# Distribution of phthisis bulbi and status of fellow eyes at a tertiary eye-care centre in Nigeria: a ten-year review

**DOI:** 10.4314/ahs.v21i1.54

**Published:** 2021-03

**Authors:** Bolajoko A Adewara, Sarat A Badmus, Olukemi T Olugbade, Edak Ezeanosike, Bernice O Adegbehingbe

**Affiliations:** 1 Department of Ophthalmology, Obafemi Awolowo University, Ile-Ife, Nigeria; 2 Department of Community Health, Obafemi Awolowo University Teaching Hospitals Complex, Ile-Ife, Nigeria; 3 Department of Ophthalmology, Alex Ekwueme Federal University Teaching Hospital, Abakaliki, Nigeria

**Keywords:** Fellow eye, Nigeria, ocular trauma, distribution, phthisis bulbi

## Abstract

**Background:**

Phthisis bulbi is an irreversible cause of visual loss with insufficient evidence about its aetiology and status of patients' fellow eyes.

**Objectives:**

To identify the distribution of patients with phthisis bulbi and determine the status of their fellow eyes at Obafemi Awolowo University Teaching Hospitals Complex, Ile-Ife, Nigeria.

**Methods:**

We analysed data retrospectively retrieved from medical records of patients diagnosed with phthisis bulbi at initial clinic visit from January 2008 to December 2017. Information abstracted included biodata, laterality of phthisical eye, duration and aetiology of phthisis bulbi, visual acuity, and morbidities present in fellow eyes.

**Results:**

Seventy-nine patients presented with unilateral phthisis bulbi. The mean age was 51±21.2 years and forty (50.6%) were males. The commonest aetiologies of phthisis bulbi were trauma 37 (46.8%), infection 17 (21.5%) and uveitis/inflammation 11 (13.9%). Seventy (88.6%) patients had morbidities in their fellow eye such as glaucoma 26 (32.9%), refractive errors 23 (29.1%) and cataract 22 (27.9%). Forty (50.6%) patients were either visually impaired or blind in their fellow eye (p=0.001).

**Conclusion:**

The commonest cause of phthisis bulbi was trauma. Approximately nine out of ten patients had ocular morbidities in their fellow eye. A thorough follow-up of patients with phthisis bulbi is recommended.

## Introduction

Phthisis bulbi is an end-stage ocular disease characterized by atrophy, shrinkage, and disorganization of the eyeball and its intraocular contents.^1^, ^2^ This usually results in visual loss as well as ocular disfigurement.^3^–^5^ The treatment of phthisis bulbi includes care of the fellow eye.^2^

On a global scale, available data on phthisis bulbi are mostly from pathologic studies of surgical eye specimens, 6–8 and subsets of patients with conditions such as penetrating eye injuries,^9^ retinoblastoma^1^, ^10^ and uveitis. 11 Furthermore, reports on causes of phthisis bulbi due to trauma,^4^ surgery,^12^ infection,^13^ inflammation,^11^ malignancy,^1^ and retinal detachment^12^ have been documented in previous research. However, the evidence is insufficient on the proportion of preventable causes responsible for this eye condition with few studies referring to the condition of the fellow eyes of patients with phthisis bulbi.^1^, ^8^

In sub-Saharan Africa, the prevalence of blindness from phthisis bulbi ranges from 4 – 19%.^3^, ^14^, ^15^ In Nigeria, phthisis is the seventh leading cause of blindness (0.1%) and severe visual impairment (0.007%).^16^ However, there is insufficient data on the distribution, the pattern of presentation of phthisis bulbi among general eye patients, and the status of their fellow eyes in sub-Saharan Africa and Nigeria.

The objective of this study was to determine the prevalence, distribution, and clinical presentation of phthisis bulbi in a tertiary eye-care setting, with emphasis on the aetiology of phthisis bulbi, as well as the visual and ocular status of their fellow eyes. This will give insight into the need for the prevention of visual impairment and blindness in the only eyes of affected individuals.

## Methods

We conducted a retrospective review of clinical records of patients who presented with phthisis bulbi at their initial visit to the Eye-care centre of the Obafemi Awolowo University Teaching Hospitals Complex (OAUTHC), Ile-Ife, Nigeria between January 2008 and December 2017. Ethical approval for the study was obtained from the hospital's Ethics and Research Committee.

Phthisis bulbi was defined as the presence of a soft and shrunken globe with evidence of structural disorganization. 11 Socio-demographic and clinical data abstracted from clinical records included gender, age, occupation, the duration of phthisis bulbi, laterality of phthisical eye, aetiology of phthisis bulbi, presenting distant visual acuity of both eyes and ocular morbidities present in their fellow eyes.

### Characteristics of variables

The age at presentation was initially categorized according to age groups and thereafter dichotomised into two groups. “Children and young adults” were defined as persons less than 45 years, and “middle-aged and elderly” defined as 45 years and older. The International Classification of Diseases 11 (2018) classification of distant visual impairment was used as a basis for defining the following:^17^

“Normal vision” as visual acuity better than or equal to 6/12 (≥ 6/12);

“Visual impairment” as visual acuity worse than 6/12 (< 6/12) or equal to 3/60;

“Blindness” as visual acuity worse than 3/60 (< 3/60). Data analysis was conducted using IBM Statistical Product and Service Solutions (SPSS) Statistics for Windows, version 25.0 (IBM Corp., Armonk, N.Y., USA). Categorical variables were analysed using chi-square tests. A p-value of less than 0.05 was considered statistically significant.

## Results

Of 19,852 clinical records for eye patients with different morbidities presenting for an initial visit to the facility during the study period, 79 patients were diagnosed with phthisis bulbi. This number accounted for a hospital prevalence of 0.4 % (79/19,852). All the patients had a unilateral phthisis bulbi (79 eyes) and all their clinical records were reviewed. There were 40 (50.6%) males and 39 (49.4%) females with a male to female ratio of approximately 1:1.

**Figure 1:**
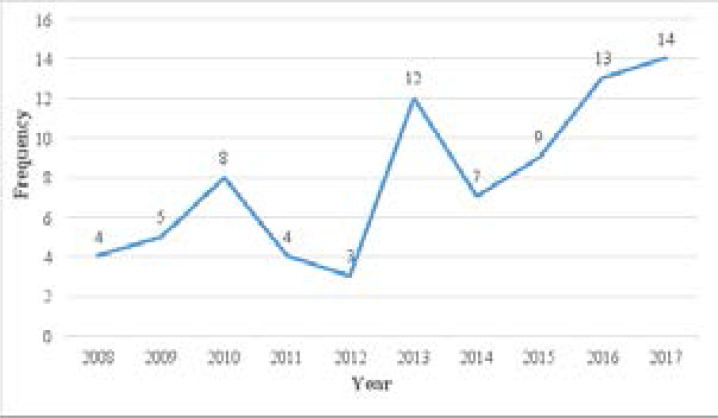
Distribution and trend of patients with phthisis bulbi, 2008 – 2017.

The prevalence over the study period was shown to intermittently peak in 2010 (n=8, 10.1%), 2013 (n=12, 15.2%) and 2017 (n=14, 17.7%) (Figure 1). The mean age at presentation was 51 ± 21.2 years with an age range of 6 months to 90 years. The mean duration of the phthisis bulbi was 12 ± 13.5 years with a range of 1 month to 60 years. Phthisis bulbi was most prevalent in the age range of 70 years and older (n=21, 26.6%) and least prevalent in the age range of 0 to 9 years (n=2, 2.5%). Of the patients reviewed, most were traders (n=22, 27.8%) and farmers (n=14, 17.7%) (Table 1).

**Table 1:** Socio-demographic and clinical characteristics of 79 patients with phthisis bulbi

	Frequency (%)
**Characteristics**	N = 79 (100)
**Gender**	
**Male**	40 (50.6)
**Female**	39 (49.4)
**Age (years)**	
**0 -9**	2 (2.5)
**10 – 19**	4 (5.1)
**20 – 29**	10 (12.7)
**30 – 39**	7 (8.9)
**40 – 49**	11 (13.9)
**50 – 59**	13 (16.5)
**60 – 69**	11 (13.9)
**70 and above**	21 (26.6)
**Occupation**	
**Trading**	22 (27.8)
**Farming**	14 (17.7)
**Schooling**	12 (15.2)
**Artisan**	9 (11.4)
**Retired**	7 (8.9)
**Civil servant**	4 (5.1)
**Teaching**	2 (2.5)
**Others**	9 (11.4)
**Laterality of phthisical eye**	
**Right**	43 (54.4)
**Left**	36 (45.6)
**Laterality of blindness (VA < 3/60)**	
**Unilateral**	69 (87.3)
**Bilateral**	10 (12.7)

VA: Visual acuity

Overall, the commonest occupation in both males and females was farming (n=10, 12.7%) and trading (n=18, 22.8%) respectively.

In the phthisical eyes, 71 (89.9%) had no perception of light, four (5.1%) had perception of light, and four (5.1%) had hand motion vision. The most common etiological factor responsible for phthisis bulbi was trauma (n=37, 46.8%), followed by infection (n=17, 21.5%) and uveitis/inflammation (n=11, 13.9%). Trauma was more common in males (n=23, 29.1%) than females (n=14, 17.7%), while infection, (females, n = 10, 12.7%; males, n = 7, 8.9%) and uveitis/inflammation (females, n = 8, 10.1%; males, n = 3, 3.8%) were more common in females than males (Table 2).

**Table 2:** Aetiology of phthisis bulbi in 79 patients by sex

		Sex		

	All patients	Male	Female	
Aetiology	N (%)	n (%)	n (%)	p-value *α*
Trauma	37 (46.8)	23 (29.1)	14 (17.7)	0.448
Infection	17 (21.5)	7 (8.9)	10 (12.7)	0.379
Uveitis/Inflammation	11 (13.9)	3 (3.8)	8 (10.1)	0.095
Post cataract surgery	4 (5.1)	3 (3.8)	1 (1.3)	0.317
Post couching	4 (5.1)	2 (2.5)	2 (2.5)	0.979
Chronic retinal detachment	4 (5.1)	2 (2.5)	2 (2.5)	0.979
Retinoblastoma	2 (2.5)	0 (0.0)	2 (2.5)	0.147
All	79 (100.0)	40 (50.6)	39 (49.4)	

α χ^2^ tests

In the fellow eyes, the common ocular morbidities were glaucoma (n=26, 32.9%), refractive errors (n=23, 29.1%) and cataract (n=22, 27.9%) (Table 3). Identified causes of blindness in their fellow eyes were glaucoma (n=3, 3.8%), corneal opacity (n=2, 2.5%), panuveitis (n=1, 1.3%), ruptured globe (n=1, 1.3%), retinoblastoma (n=1, 1.3%), chronic retinal detachment (n=1, 1.3%) and retinitis pigmentosa (n=1, 1.3%).

**Table 3:** Distribution of ocular morbidities in 79 fellow eyes of patients with phthisis bulbi

Ocular morbidity	Fellow eye (%)
**Glaucoma**	26 (32.9)
**Refractive error**	23 (29.1)
**Cataract**	22 (27.9)
**Nasal pterygium**	7 (8.9)
**Allergic conjunctivitis**	5 (6.3)
**Corneal opacity**	3 (3.8)
**Lens dislocation**	3 (3.8)
**Presbyopia**	3 (3.8)
**Panuveitis**	2 (2.5)
**Macular scar**	2 (2.5)
**Ruptured globe**	1 (1.3)
**Retinoblastoma**	1 (1.3)
**Chronic retinal detachment**	1 (1.3)
**Ischemic CRVO**	1 (1.3)
**Retinitis pigmentosa**	1 (1.3)
**Age related macular degeneration**	1 (1.3)
**Cystoid macular oedema**	1 (1.3)
**Pinguecula**	1 (1.3)
**Chorioretinal scar**	1 (1.3)
**Diabetic retinopathy**	1 (1.3)
**Normal eye**	9 (11.4)

CRVO: Central retinal vein occlusion

Our findings showed that based on visual acuity, there were higher proportions of visually impaired or blind fellow eyes among patients who were 45 years or older (n=31, 39.2%; p=0.015), those who had non-traumatic causes of phthisis bulbi (n=26, 32.9%; p=0.033) and those with ocular morbidities in their fellow eye (n=40, 50.6%; p=0.001)(Table 4).

**Table 4:** Characteristics of 79 patients with unilateral phthisis bulbi based on visual acuity in the fellow eye

		Normal vision	Visual impairment and blindness	

	All patients	(VA≥6/12)	(VA<6/12)	
Characteristics	N = 79 (100)	n = 39 (%)	n = 40 (%)	p-value*α*
**Gender**				
Male	40 (50.6)	22 (27.8)	18 (22.8)	0.311
Female	39 (49.4)	17 (21.5)	22 (27.8)	
**Age (years)**				
< 45	28 (35.4)	19 (24.1)	9 (11.4)	0.015*
≥ 45	51 (64.6)	20 (25.3)	31 (39.2)	
**History of trauma**				
Present	37 (46.8)	23 (29.1)	14 (17.7)	0.033*
Absent	42 (53.2)	16 (20.3)	26 (32.9)	
**Ocular morbidity** **in the fellow eye**				
Present	70 (88.6)	30 (38.0)	40 (50.6)	0.001*
Absent	9 (11.4)	9 (11.4)	0 (0.0)	

VA: Visual acuity

α χ^2^ tests

* Significant

## Discussion

The prevalence of phthisis bulbi in this study was low amongst general eye patients attending the Eye-care centre of a tertiary hospital in Ile-Ife, Nigeria. This result contributes to the data on the prevalence of phthisis bulbi in this study population. However, this result was difficult to compare with previous studies^7^, ^9^, ^11^ because of the differences in the study populations. The value was lower compared to 19% found in patients attending a uveitis clinic,^11^ 11.7% among surgical eye specimens,^7^ and 6.1% among patients with penetrating eye injuries.^9^ The dissimilarities in the prevalence of phthisis bulbi could be a result of etiological differences among the study populations.

In this study, about two-thirds of the phthisis bulbi due to trauma were among males. This may be because occupation related injuries are associated with occupations such as farming which was the commonest occupation among males in this study.^18^ Though some researchers reported a male preponderance among patients with phthisis bulbi similar to our study, the relationship with occupation was not evaluated.^8^, ^9^, ^11^

Phthisis bulbi was more common among middle-aged and elderly patients compared to children and young adults. The cause of more patients presenting in middle age or old age may be due to disorders of the fellow eye, which compels them to visit the hospital. However, young people with good vision in the fellow eye may not visit the hospital especially in areas with lack of resources and other barriers to health care. In addition, with increasing age, the chances of experiencing trauma or disease in the eye may increase, as the available time for this to occur is more. Furthermore, some causes of phthisis bulbi in the younger age group such as developmental eye defects were not found in this study, though there were a few cases of retinoblastoma.

About a third of the patients who were 45 years or older were found to be visually impaired or blind in their fellow eye. This may be because older patients are more predisposed to age-related ocular diseases such as glaucoma and cataract, which were found to be more common in this study. Patients could also have presented primarily because of the reduced vision in their fellow eyes.

A study by Tan et al,^11^ in the United Kingdom, reported the age range at the onset of phthisis to be 17 – 97 years among patients in a uveitis clinic which was higher than 2–16 years reported in eight patients with penetrating eye injuries by Coskun et al,^9^ in Turkey. These findings suggest that phthisis bulbi is less common in children and aetiological factors such as trauma, uveitis, or retinoblastoma in the populations being studied may play an important role in the age distribution of patients with this disease.

Trauma and infection were the most common causes of phthisis bulbi in our study. This finding indicates that more than two-thirds of the aetiology of phthisical eyes were preventable. This was corroborated by reports from de Gottrau et al,^6^ Kitzmann et al,^7^ and Tan et al,^11^ who also found trauma to be the commonest cause of phthisis bulbi.

Incidentally, our study also showed that visual impairment and blindness were significantly higher in fellow eyes of patients with phthisis bulbi from non-traumatic causes (p = 0.033). Hence, non-traumatic causes may more likely predispose to ocular morbidities in fellow eyes of patients with phthisis bulbi.

Expectedly, all the eyes with phthisis bulbi were blind but only about a tenth of the patients had fellow eyes without any ocular morbidities. This highlights the fact that all patients with phthisical eyes would require care of the fellow eye because it is the only eye. The most common ocular morbidities found in fellow eyes of patients with phthisis bulbi were glaucoma, refractive errors, and cataract. Furthermore, the fellow eye in more than fifty percent of patients, was either visually impaired or blind. Fortunately, most of the conditions found in the fellow eyes were either curable or vision could be maintained with proper care and follow up. The presence of ocular morbidities in their fellow eyes resulted in the increased proportion of patients with visual impairment or blindness in these eyes (p = 0.001). There is a paucity of literature on ocular morbidities in fellow eyes of patients with phthisis bulbi with which to make comparisons, especially as some data are based on pathological studies of surgical eye specimens.^6^–^8^ However, Kashyap et al,^10^ reported findings of retinoblastoma in fellow eyes of 15 (83.3%) of their patients and Shah et al,^19^ reported a case of myofibroblastic tumour in the fellow eye of a patient with phthisis bulbi. Tan et al,^11^ also reported the presence of penetrating eye injuries in eight (12.5%) fellow eyes and retinal detachment in three (4.7%) fellow eyes of patients with phthisis bulbi due to sympathetic ophthalmitis. These reports call attention to the presence of potentially blinding eye conditions in the only eyes of patients with phthisis bulbi. This further emphasises the need for eye care and monitoring of the fellow eyes of these patients.

It is worth noting that despite the high prevalence of phthisis bulbi from traumatic causes in this study there was no case of sympathetic ophthalmitis found in any fellow eye. This important finding may indicate the possibility of a reduced risk of sympathetic ophthalmitis in this study population possibly due to racial differences. The retrospective methodology was a limitation because the compilation of data was based only on patients' documented past ocular histories and clinical findings. This methodology limited our ability to determine the number of cases who had penetrating or non-penetrating trauma. Secondly, this study was carried out in a hospital setting, so the prevalence may not represent the data of the population. A subsequent prospective or community-based study would be able to address these limitations.

## Conclusions and recommendations

This study found that, though an uncommon disease, about one-fifth of patients with phthisis bulbi reviewed were elderly individuals. The commonest cause of phthisis bulbi identified in our study population was trauma and about 9 out of 10 patients had ocular morbidities in their fellow eye, of which more than half of the eyes reviewed were either visually impaired or blind. This study also showed that patients who were forty-five years or older, those who had non-traumatic causes of phthisis bulbi, and patients with ocular morbidities in their fellow eye, had a significantly higher proportion of fellow eyes that were either visually impaired or blind.

We recommend regular follow up of patients with phthisis bulbi and the identification and prompt care of morbidities in their fellow eyes as an essential part of their treatment. This would optimize their vision, prevent blindness, and thus improve the quality of life especially of patients forty-five years or older and those with non-traumatic causes.
